# Foraging Bumblebees Selectively Attend to Other Types of Bees Based on Their Reward-Predictive Value

**DOI:** 10.3390/insects11110800

**Published:** 2020-11-13

**Authors:** Jose E. Romero-González, Amanda L. Royka, HaDi MaBouDi, Cwyn Solvi, Janne-Tuomas Seppänen, Olli J. Loukola

**Affiliations:** 1 Department of Biological and Experimental Psychology, School of Biological and Chemical Sciences, Queen Mary University of London, London E1 4NS, UK; j.e.romerogonzalez@qmul.ac.uk (J.E.R.-G.); A.L.Royka@sms.ed.ac.uk (A.L.R.); h.maboudi@sheffield.ac.uk (H.M.); c.solvi@qmul.ac.uk (C.S.); 2 Department of Computer Science, University of Sheffield, Sheffield S1 4DP, UK; 3 Open Science Centre, University of Jyväskylä, P.O. Box 35, 40014 Jyväskylä, Finland; janne.t.seppanen@jyu.fi; 4 Department of Ecology and Genetics, University of Oulu, P.O. Box 3000, 90014 Oulu, Finland

**Keywords:** bees, behavioral flexibility, insects, selective attention, social learning

## Abstract

**Simple Summary:**

Social information use is a widespread phenomenon in animals affecting various important aspects of behavior. Animals observe and copy the behavioral patterns of conspecifics and other species on the same trophic level in their own decision-making. Copying others is adaptive only when it is selective, otherwise running the risk of copying bad behavior. Thus, it would be important to know when and which individuals should be copied. We showed that bumblebees *Bombus terrestris* learn to attend to specific types of bees based on their learned value as information-providers about reward. Learned information was transferred across foraging contexts, representing a useful pathway for bees to acquire relevant information about unfamiliar floral resources that goes beyond conspecific social information use, extending to heterospecifics. Our findings indicate that the transmission of social information across species can be highly selective in response to learned value of the information provider, plausibly making the phenomenon adaptive.

**Abstract:**

Using social information can be an efficient strategy for learning in a new environment while reducing the risks associated with trial-and-error learning. Whereas social information from conspecifics has long been assumed to be preferentially attended by animals, heterospecifics can also provide relevant information. Because different species may vary in their informative value, using heterospecific social information indiscriminately can be ineffective and even detrimental. Here, we evaluated how selective use of social information might arise at a proximate level in bumblebees (*Bombus terrestris*) as a result of experience with demonstrators differing in their visual appearance and in their informative value as reward predictors. Bumblebees were first trained to discriminate rewarding from unrewarding flowers based on which type of “heterospecific” (one of two differently painted model bees) was next to each flower. Subsequently, these bumblebees were exposed to a novel foraging context with two live painted bees. In this novel context, observer bumblebees showed significantly more social information-seeking behavior towards the type of bees that had predicted reward during training. Bumblebees were not attracted by paint-marked small wooden balls (moved via magnets) or paint-marked non-pollinating heterospecifics (woodlice; *Porcellio laevis*) in the novel context, indicating that bees did not simply respond to conditioned color cues nor to irrelevant social cues, but rather had a “search image” of what previously constituted a valuable, versus invaluable, information provider. The behavior of our bumblebees suggests that their use of social information is governed by learning, is selective, and extends beyond conspecifics.

## 1. Introduction

Animals can gather information about their environment through personal exploration or by learning from others through observation [1]. All animals are exposed to continuously changing environments where acquiring reliable information becomes crucial for ensuring survival and reproductive success [2,3,4,5]. Monitoring other individuals’ behaviors can provide useful information for decisions regarding foraging, mating, and detection of danger [3]. However, theoretical models and empirical studies suggest that using social information indiscriminately is not adaptive and may even lead to the spread of maladaptive behavior [6,7]. Therefore, animals are expected to use social information in a selective manner [5,8,9,10].

The quality or relevance of different sources of information may differ from the perspective of social information users—a variety of vertebrates have been shown to preferentially use social information from successful [11,12], experienced [13,14], dominant [15,16], older [17,18] and larger individuals [19], as well as conspecifics of a certain sex [20,21] or kinship class [22,23]. Alternatively, experience may lead animals to learn more readily from particular classes of individuals [24]. On evolutionary and adaptive grounds, animals are expected to recognize and favor social information from conspecific over heterospecific sources [25,26], but in most natural communities, heterospecifics are a prolific source of social cues that may provide relevant information [5,27,28].

The ecology of pollinating insects, such as bumblebees, offers a rich social environment that might facilitate the use of social information from conspecific and heterospecific sources [29]. Learning mechanisms based on elemental associations, either Pavlovian or operant conditioning, may account for social learning in bees [30]. It is well-established that bumblebees can learn to integrate two learned associations (second-order conditioning); they form associations between rewarding flowers and the presence of conspecifics [31] and heterospecifics [32]. Bumblebees have provided an excellent model to study the mechanisms that underlie the context dependency of social information use [10,29,33]. For example, bumblebees can learn to use or ignore conspecific social cues depending on their reliability to predict a reward [34,35]. They also tend to prefer using social over personal information, unless social information is unreliable [36]. Likewise, they are more likely to rely on social information when foraging in unpredictable environments [37], environments with patchily distributed resources [38], or when the foraging task is cognitively more difficult [39].

The vast foraging areas exploited by bumblebees incorporate social cues from a large number of pollinating insect species with overlapping ecological niches [40,41,42,43,44]. Species can differ from each other in morphology, ecology, and behavior, creating a large variation in the quality and relevance of the available social information to members of other species [5,45]. From an adaptive perspective, it is conceivable that cognitive mechanisms may allow flexible responses to such differences [46], and therefore, animals should use social information selectively, attending to the presence or behavior of those species which predictably lead to an increase in the observer’s foraging efficiency [5].

Here, we examine whether bumblebees lacking personal information about reward locations in a novel foraging context will selectively attend to the type of demonstrator that was previously associated with a predictable reward. Selective attention is the process of focusing, for a period of time, on a specific item in the environment. It plays a vital role in decision making in most animals, including many invertebrates [47,48,49,50,51]. Specifically, we experimentally test whether bumblebees can first learn to discriminate between two types of artificially-modified (paint-marked) bumblebees, based on their informative value to predict reward and, subsequently, whether they preferentially explore the immediate vicinity of one of two similarly paint-marked live bumblebees in a separate, novel foraging task.

## 2. Materials and Methods

### 2.1. Set-Up

Bumblebee (*Bombus terrestris*) foragers (14 colonies), obtained from Biobest, Belgium N.V., were used for the experiments. Forager bees used in the experiments and control experiments were randomly selected from several colonies to avoid pseudoreplication. Bumblebees and their nests were housed in two-chambered wooden boxes (l = 29.5 × w = 11.5 × h = 9.5 cm) connected to a wooden flight arena (l = 58 cm, w = 40 cm, h = 24 cm) by a Plexiglas tunnel (l = 25 cm, 3.5 × 3.5 cm). The flight arena floor was covered with a Gaussian blurred random dot pattern produced with MATLAB (MathWorks, 2015b), which provided a high contrast between the color of the bees and the background to facilitate video analysis. Three plastic sliding doors located along this corridor allowed controlled access to the arena. Before and after experiments, bees could freely forage from a mass feeder in the middle of the arena, which contained 30% (m/m) sucrose solution. Colonies were provided with 7 g commercial pollen (Koppert B.V., Berkel en Rodenrijs, The Netherlands) every two days.

### 2.2. Pre-Training of Observer Bees

Foragers from five colonies were pre-trained to forage on an array of eight plastic chips (artificial flowers; hereafter “flowers”; 2.4 × 2.4 × 0.4 cm) positioned on the back wall of the arena. The array was arranged in two rows of four flowers each, with a separation of 7.5 cm between them. To familiarize bees with the place of the rewards on the back wall, flowers were replenished (after they had been emptied by bumblebees and the bumblebees had left the flower) with rewarding 50% (m/m) sucrose solution. In this pre-training phase, all the foragers in a colony were allowed to freely feed on the array for two hours.

### 2.3. Training Observer Bees to Differentiate Model Demonstrators by Their Reliability to Predict Reward

To establish if associative mechanisms might direct bumblebees’ attention to a specific type of demonstrator based on whether they had previously predicted reward, we first had to determine if they could readily learn to discriminate model demonstrators that were visually distinct (thoraxes painted with different colors).

**Figure 1:**
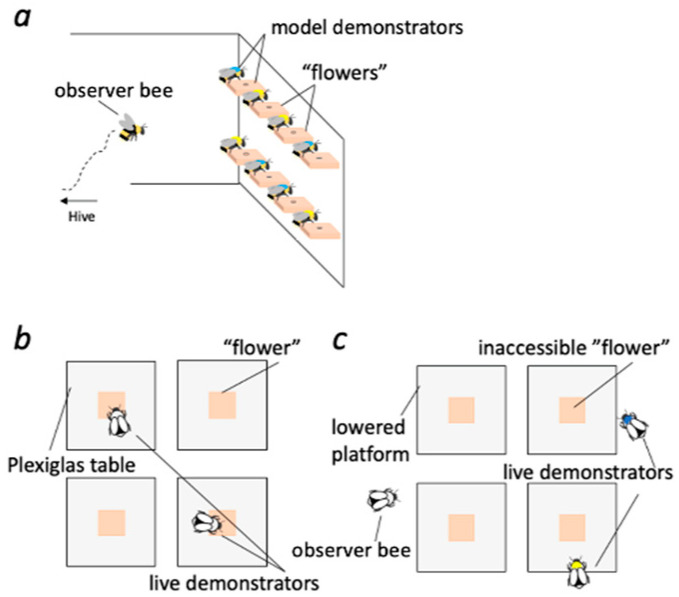
Schematic views of the arena during training and testing. (**a**) Observer bees were trained to find 50% sucrose solution on four of the eight flowers arranged on the far wall of the arena and aversive quinine solution on the other four flowers. Next to each of the flowers was placed one of two types of model demonstrator. Each model demonstrator’s thorax was painted blue or yellow. (**b**) Live demonstrators were trained to crawl underneath four Plexiglas platforms to obtain a reward on a flower. Trained individuals were then given either a blue or yellow paint mark on their thorax prior to testing the observer. (**c**) Observer bees were tested in a novel context featuring two live demonstrators. In this novel situation, the flowers were not accessible, and as a result, the live demonstrators searched the floor of the arena for reward.

To do this, all observer bees within a colony were group trained to find reward at a similar array of flowers as in the pre-training phase. However, during this training, we randomly assigned half (four) of the flowers to contain aversive saturated quinine solution (1.2 mg/mL H_2_O), while the other half (four) contained rewarding 50% (m/m) sucrose solution. On each flower, one of two types of model demonstrator bees was positioned on the side of the flower as though it was feeding (Figure 1a). The type of model demonstrator was only distinguishable with a blue or yellow color mark on their thorax. We used model demonstrators so we could control and vary the spatial location of reinforcements and demonstrators easily. Model demonstrators were made by first cooling live bumblebees until dead by placing them in a freezer at −20 °C (exposure of bumblebees to freezing temperatures results in lethargy, sleep, and then death within minutes [52]). Model demonstrators were subsequently fixed in 100% ethanol for five hours and placed in an incubator at 60 °C to dry for one hour. Their thorax was color-marked with either blue or yellow paint (0.6-cm Ø; Posca Pens, Worcester, UK) and their entire body was coated with acrylic spray (PKT24000, Plasti-kote, Wokingham, UK). This preparation method allowed the model demonstrators to be cleaned with 70% ethanol in water (*v*/*v*) between training and testing sessions. During the experiments, observer bumblebees never touched the model demonstrators. However, to ensure observer bees would not learn to follow any scent cues, model demonstrators, flowers, and the arena wall were cleaned by wiping each with tissue paper soaked in 70% ethanol in water (*v*/*v*) and subsequently wiping with dry tissue paper between each visit to the arena by an individual observer bee and before test trials.

Observer bees (*n* = 19) from two colonies were trained with yellow-marked bees (model demonstrators) pinned next to the flowers containing sucrose solution, while blue-marked model demonstrators were pinned next to the flowers containing quinine (Figure 1a). This color-reward arrangement was reversed for the remaining observer bees from three colonies (*n* = 23), controlling for potential color bias. Bees were allowed to forage freely in the arena, and training took place over two consecutive days (six sessions of 30 min each). Observer bee identity was tracked with individual number tags (Opalithplättchen, Warnholz & Bienenvoigt, Ellerau, Germany) attached to the top of their thorax by Super Glue Gel (Loctite, OH, USA).

### 2.4. Confirming Individual Learning of Observer Bees

Bumblebees identified as regular foragers (feeding from the flowers, transporting their crop load to the nest, and returning to the flowers several times and taking less than 10 min per trip) during the training phase were individually evaluated in an unrewarded trial to confirm they had learned to differentiate the model demonstrators in terms of their reliability to predict reward. We used the same wall set-up as in the training phase (Materials and Methods, Section 2.2), but all the flowers contained water, and the model demonstrator placements were randomly re-assigned. Each observer was tested individually by releasing them into the arena and recording the first twenty landings. An observer was considered to have learned and was used in the main experiment if the proportion of correct choices, i.e., landings on flowers occupied by the model demonstrator type that previously predicted reward, was at least 0.75. If observer bees did not land on any flower after 5 min, they were removed from the arena and not used for the remainder of the experiments.

Twenty-three observer bees from five colonies (colony 1:5, colony 2:5, colony 3:5, colony 4:4, and colony 5:4) were confirmed to be regular foragers that had learned the association between model demonstrator color cue and reward (see DEMONSTRATOR TYPE DISCRIMINATION MODEL below), and were used in the experimental trials (see Section 2.6).

### 2.5. Training of Live Demonstrators

Forager bees from one colony (colony 6) were pre-trained to find a reward, 50 μL of 50% (m/m) sucrose solution, on each of the four flowers on the floor of the arena (Figure 1b). Bees were allowed to forage freely as a group from the flowers for 1 h (ad libitum reward as the experimenter refilled the flowers after they were emptied and the bees left the flower). After pre-training, the flowers were gradually positioned under transparent acrylic platforms (8 × 8 × 1 cm). When the flowers were positioned entirely under the acrylic platforms, the bees had to crawl underneath to obtain the reward. The positioning of the flowers under the platforms was done to ensure that live demonstrators would spend extended periods of time during the trials searching the floor for the reward. Pilot experiments revealed that easy access to the reward during training caused live demonstrators to become de-motivated quickly and fly around the arena soon after discovering no reward was available. Successfully trained individuals were pseudo-randomly (flip of a coin) given either a blue or yellow mark equivalent to the marks displayed by the model demonstrators. Similar to model demonstrators, the color marks were applied to their thorax while holding the bees carefully with forceps.

### 2.6. Testing Observer Bees in a Novel Context with Live Demonstrators

Twenty-three successfully-trained observer bees (out of 42 bees; see Section 2.3 and Section 2.4) from five colonies (colony 1:5, colony 2:5, colony 3:5, colony 4:4, and colony 5:4) were tested on whether the information they had learned previously (reliability of model demonstrator type to predict reward) influenced their social behavior in a novel context with live demonstrators. Observer bees were tested in the same set-up we used to train the live demonstrators. However, the Plexiglas platforms were lowered to 0.5 cm above the floor so that bees could not crawl underneath to reach the flowers (Figure 1c). Because the live demonstrators had been trained to crawl under the platform to receive the reward, during these trials, they spent long periods of time on the floor searching for the reward. After two live demonstrator bees (one of each color) were let into the arena, one observer bee was allowed to enter the arena. Observer bees were given up to 5 min to gain familiarity with the new set-up. Following the first instance where the bee displayed a slow side-to-side flight within several centimeters of a demonstrator (indicating that she was paying attention to the live demonstrator), the trial began and lasted 5 min (see INFORMATION ATTENTION MODEL and IN-FLIGHT DECISION MODEL below). This behavioral criterion for the start of data collection was verified with each video. If observer bees did not attend to the live demonstrators’ presence after 5 min, they were returned to the nest and not tested again.

### 2.7. Control Groups

**Figure 2:**
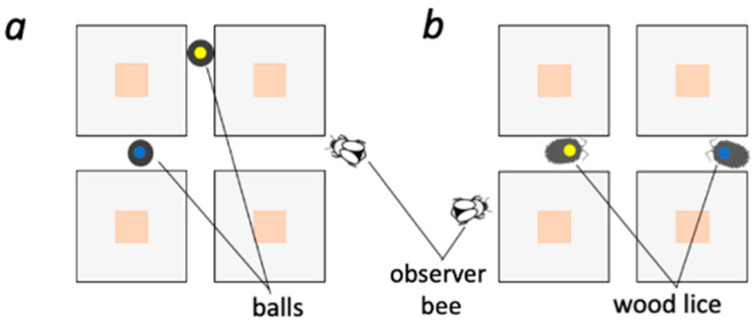
Bumblebees do not simply attend to the color cues in a novel scenario. (**a**,**b**) After two new groups of bumblebees were trained on the horizontal wall situation (Figure 1a), they were then tested in a novel context featuring two demonstrators, either small wooden balls (**a**) or a non-nectivorous species (woodlice; **b**). In this novel situation, the balls were moved via internal magnets and magnets under the arena floor. The woodlice roamed around the arena floor spontaneously.

Bumblebees readily form associations between flower colors and the presence of conspecifics [53,54,55,56]. Therefore, rather than selectively attending to social information from artificially-modified bumblebees differing in their informative value, our observer bumblebees may have learned to associate only the color on the demonstrator’s thorax with reward or punishment. To rule this out, a group of bumblebees, trained in the same way as the previous group of observer bees (Section 2.3 above, Figure 1a), was tested in the same novel foraging context (Section 2.6 above, Figure 1c). Here, however, live demonstrators were replaced by either two black wooden balls (1.5 cm^3^) with 0.6-cm ∅ color paint marks (one yellow and one blue) that were moved around the arena, mimicking the speed and movements of walking bumblebees around the four Plexiglas tables covering the flowers (within 2 cm from the edge of any of the tables), via an attached magnet manipulated from the underside (outer surface) of the arena floor, or replaced by two woodlice with 0.6-cm ∅ paint marks that roamed freely around the arena floor. These groups consisted of 40 foragers randomly selected from eight different colonies (wooden balls—colonies 7–10: 5 observer bees each; woodlice—colonies 11–14: 5 observer bees each; Figure 2a,b; see DEMONSTRATOR TYPE DISCRIMINATION MODEL below).

Another group of bees was evaluated to rule out an innate bias towards either of the color cues displayed by the live demonstrators (see control group in INFORMATION ATTENTION MODEL below). This control group consisted of 13 foragers randomly selected from the six experimental colonies (colony 1:3, colony 2:2, colony 3:2, colony 4:2, colony 5:2, and colony 6:2). These foragers were pre-trained in the same way as the experimental group, i.e., they learned to feed upon a horizontal array of eight flowers. However, for the control group, no model demonstrators were present on the flowers during training. Individual observer bees were tested in the novel context with two differently color-marked live demonstrators, as described for the experimental group in Section 2.6 above.

### 2.8. Behaviors Measured in Test and Control Trials

All trials were recorded from above the arena with an iPhone 6 camera (Apple, CA, USA) with a recording frame rate of 240 fps. We measured *instances of proximity* with live demonstrators, where the observer bee and live demonstrator were both on the floor of the arena and within one body length of each other. Close interaction between the observer bee and the live demonstrator ensured that the observer bee detected the color of a 0.6-cm Ø mark, the diameter of the painted marks on our demonstrators [57]. We also measured if the observer bee hovered in flight near a demonstrator with their body positioned in the direction of (facing) the live demonstrator (for at least 0.1 s) and landed next to the live demonstrator (*choosing the live demonstrator*) or flew away without landing next to the live demonstrator (*rejecting the live demonstrator*). These were divided into correct decisions (*choosing the reward-predicting live demonstrator* or *rejecting the punishment-predicting live demonstrator*) and incorrect decisions (*choosing the punishment-predictive live demonstrator* or *rejecting the reward-predictive live demonstrator*) (see IN-FLIGHT DECISION MODEL below). The experimenter analyzing the videos was blind to the color each bee had been trained to and whether the bee had been trained at all.

### 2.9. Analysis

All statistical analyses were conducted with R version 3.4.3 [58]. Generalized linear mixed-effects models (GLMMs; ‘glmer’ function in package lme4 [59]) were used. Four models were derived. The relative influence of each observation was adjusted in the COLOR DISCRIMINATION MODEL, the INFORMATION ATTENTION MODEL, and the IN-FLIGHT DECISION MODEL by using ‘weights’ function. The fitted values were transformed into a probability scale by using the ‘plogis’ function in the DEMONSTRATOR TYPE DISCRIMINATION MODEL and the INFORMATION ATTENTION MODEL. In the INFORMATION ATTENTION MODEL, the Proximity Index was scaled to [0, 1] by using a using ‘rescale’ function to fit the data better with logit link function (binomial family). In addition, zero values were not included because they indicate that an observer bee spent no time in proximity with either live demonstrator bee.

#### 2.9.1. Do Bumblebees Preferentially Attend to Other Bees Rather than Simple Color Cues or Generic Insect-Shaped Cues?

**The DEMONSTRATOR TYPE DISCRIMINATION MODEL** tested whether bumblebees attended to specific types of other bees rather than simple color cues on objects or generic insect-shaped cues. The following formula was used: glmer(proximity instances~demonstrator type + (1|colony) + (1|Bee identity), family = ‘poisson’).

#### 2.9.2. Discriminating between Model Demonstrators of Differing Visual Appearance and Informative Value as Reward Predictors

**The COLOR DISCRIMINATION MODEL** tested whether the observer bees were more likely to land on flowers next to the type of model demonstrators in the test phase (Section 2.4. above) that had previously been next to rewarding flowers in the training phase (Section 2.3. above) and tried to determine whether the color on the thorax of the demonstrator affected observer bumblebees’ performance. The following formula was used: glmer(performance (proportion of correct landings)~1 + rewarding color + (1|colony), family = ‘binomial’, weights = total number of landings on flowers).

#### 2.9.3. The Effect of Experience on Observer Bees’ Attention to Live Demonstrators Differing in Their Informative Value as Reward Predictors

**The INFORMATION ATTENTION MODEL** tested whether the observer bees selectively attended to the type of demonstrator that was previously associated with reward and whether that differed between the trained and control observer bees.

To control for the amount of time that live demonstrators spent on the ground of the arena (i.e., available for the observer bees to observe and interact with), we normalized the proximity instances into an index:

$$Proximity\;\mathrm{Index}=\;\frac{\frac{i_{\mathrm{Op}}^{+}}{t_{\mathrm{Dg}}^{+}}\;-\;\frac{i_{\mathrm{Op}}^{-}}{t_{\mathrm{Dg}}^{-}}}{\frac{i_{\mathrm{Op}}^{+}}{t_{\mathrm{Dg}}^{+}}\;+\;\frac{i_{\mathrm{Op}}^{-}}{t_{\mathrm{Dg}}^{-}}}$$

where, $i_{\mathrm{Op}}^{+}$ and $i_{\mathrm{Op}}^{-}$ are the instances of proximity that the observer had with the demonstrators previously associated with reward and punishment, respectively, and $t_{\mathrm{Dg}}^{+}$ and $t_{\mathrm{Dg}}^{-}\;$ are the total time these live demonstrators spent on the ground of the arena. The control group had no experience with the demonstrators and, thus, were used to assess whether bumblebees preferred either colored bee. Therefore, for the control group, $i_{\mathrm{Op}}^{+}$ and $t_{\mathrm{Dg}}^{+}$ correspond to the times relating to the demonstrator displaying the blue mark, whereas $i_{\mathrm{Op}}^{-}$ and $t_{\mathrm{Dg}}^{-}$ correspond to the times obtained for the demonstrator displaying the yellow mark. In the indices, a positive value (maximum +1) equates to a preference of the observer bees for the demonstrator that previously predicted reward, whereas a negative value (minimum −1) equates to a preference for the demonstrator that previously predicted punishment. Similarly, an index near equal to zero indicates the observer bees interacted with both live demonstrators equally relative to the amount of time each was available.

The following formula was used: glmer(proximity Index (rescaled to 0,1)~treatment + (1|colony), family = ‘binomial’, weights = total number of proximity instances).

#### 2.9.4. The Effect of Live Demonstrators’ Informative Value as Reward Predictors on in-Flight Decisions

**The IN-FLIGHT DECISION MODEL** tested whether the hovering time of a bee in flight near a demonstrator with their body positioned in the direction of (facing) the demonstrator (for at least 0.1 s) affected the observer’s decisions (choosing or rejecting the demonstrator). The following formula was used: glmer(performance (proportion of correct decisions)~hovering time before decision + (1|colony), family = ‘binomial’, weights = total number of decisions).

## 3. Results

### 3.1. Do Bumblebees Preferentially Attend to Other Bees Rather Than Simple Color Cues or Generic Insect-Shaped Cues?

**The DEMONSTRATOR TYPE DISCRIMINATION MODEL** shows that observer bumblebees had significantly less proximity instances with balls (4 out of 20 bees, GLMM (*Proximity instances*); estimate −2.42, SE = 0.75, t = −3.24, *p* = 0.001) or woodlice (2 out of 20 bees, Estimate = −4.36, SE 0.98, t = −4.47, *p* < 0.001) of any color, compared to live bumblebees (18 out of 23 bees, estimate 1.16, SE 0.42, t = 2.77, *p* < 0.006). As so few bees attended to balls or woodlice, further statistical analysis of color preferences is not warranted.

### 3.2. Discriminating between Model Demonstrators of Differing Visual Appearance and Informative Value as Reward Predictors

**The DEMONSTRATOR COLOR DISCRIMINATION MODEL** shows that observer bees landed preferentially on flowers next to the model demonstrators that had previously predicted reward (GLMM: 95% confidence interval (CI) = 0.70 (0.63 to 0.77), *n* = 42 bees, *p* < 0.001). There was no difference between the preference of observer bees for blue- or yellow-painted model demonstrators (GLMM: 95% confidence interval (CI) = 0.48 (0.35 to 0.62), *n* = 42 bees, *p* = 0.78).

### 3.3. The Effect of Experience on Observer Bees’ Attention to Live Demonstrators Differing in Their Informative Value as Reward Predictors

**Figure 3:**
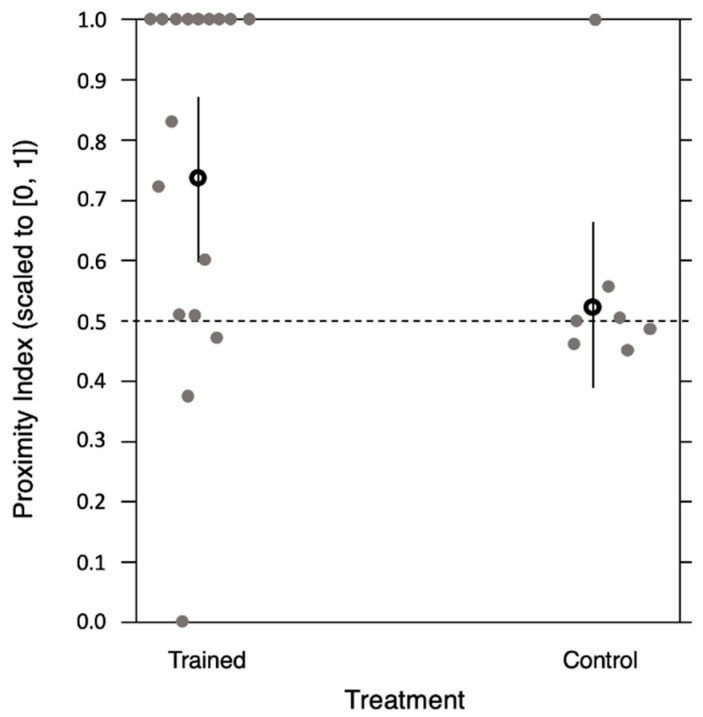
Selective attention to live demonstrators was not influenced by amount of time demonstrators were on the floor. Proximity index (scaled to [0, 1]) represents the total number of proximity instances in relation to the total amount of time each live demonstrator spent on the floor and, therefore, was available for the observer to follow. Trained observer bees selectively attended to the type of live demonstrator that was previously associated with reward and control bees trained on the vertical flowers without the presence of any demonstrators did not show preference for either of the different artificially-modified (yellow- or blue-painted thorax) demonstrators. Open circles represent model estimates (0.73 for trained bees and 0.53 for control bees) for both and error bars represent confidence levels at 95% (0.60 to 0.87 for trained bees and 0.39 to 0.67 for control bees); each grey circle represents Proximity indexes (scaled to [0, 1]) for individual bees. The horizontal dashed line indicates the chance level (no preference).

**The INFORMATION ATTENTION MODEL** shows that trained observer bees selectively attended to the type of demonstrator that was previously associated with reward (GLMM: 95% confidence interval (CI) = 0.73 (0.60 to 0.87), *n* = 17, *p* = 0.001) and control bees trained on the vertical flowers without the presence of any demonstrators did not show preference for either of the different artificially-modified (yellow- or blue-painted thorax) demonstrators (95% confidence interval (CI) = 0.53 (0.39 to 0.67), *n* = 7, *p* = 0.80, Figure 3).

### 3.4. The Effect of Live Demonstrators’ Informative Value as Reward Predictors on In-Flight Decisions

**Figure 4:**
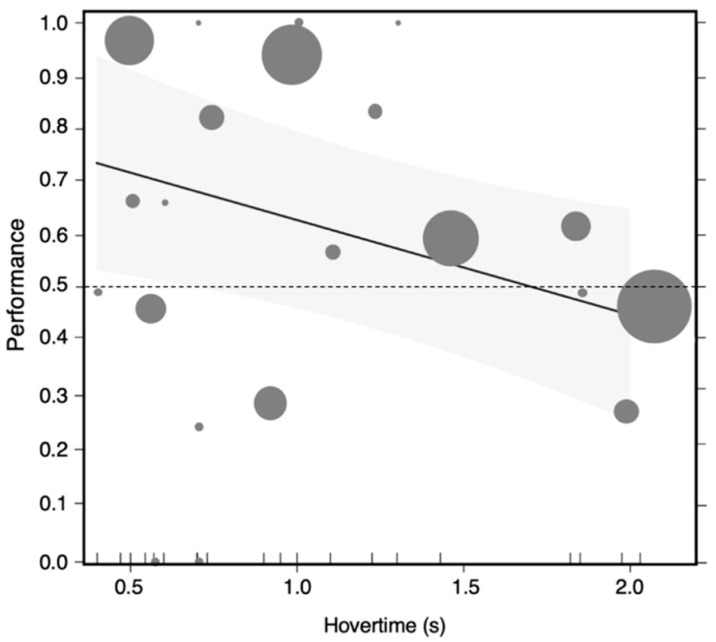
In-flight decision performance was influenced by decision time. Observer bees made significantly more correct in-flight decisions (choosing the reward-predicting live demonstrator or rejecting the punishment-predicting live demonstrator) than chance level (50%). The proportion of correct decisions decreased over hovering time. The figure shows the predicted levels of performance (proportion of correct decisions) and its 95% confidence band for the sample values of hovering time before decision. Each circle represents in-flight decision performances and mean hovering times before decision for individual bees. The size of the circle indicates the total number of decisions for individual bees. The horizontal dashed line indicates the chance level (50%).

**The IN-FLIGHT DECISION MODEL** shows that observer bees made significantly more correct in-flight decisions (choosing the reward-predicting demonstrator or rejecting the punishment-predicting demonstrator) in general (GLMM; estimate = 1.28, SE = 0.52, z = 2.47, *p* = 0.013) compared to chance (50 %), but their performance (proportion of correct decisions) decreased over hovering time (estimate = −0.75, SE = 0.31, z = −2.40, *p* = 0.017, Figure 4).

## 4. Discussion

Our results confirm that bumblebees can discriminate between different types of artificially-modified model bumblebees via learned associations between distinctive visual cues, characterizing such models and the models’ relative informative value to predict reward.

Observer bumblebees did not follow irrelevant asocial or irrelevant social cues, but rather had a specific “search image” of what previously constituted a valuable, versus invaluable, information provider. The behavior of bumblebees suggests that their use of social information is governed by learning, is selective, extends to visually novel characteristics of conspecifics, and is generalized to novel foraging contexts, suggesting this selectivity may extend to other species also.

Our results also suggest that the more time bumblebees took to make a decision, the less accurate they were in that decision. This result was surprising as earlier experiments in bumblebees [60,61] have found support for the presence of a trade-off between speed and accuracy. One possible explanation for our result is that bees that had a better long-term memory and formed stronger associations between the color and the reward/punishment-predictive demonstrators were also making faster decisions, while those that had poorer memories of the association took longer to decide.

### 4.1. Benefits of Using Social Information

Our results are in line with the social learning strategy, where using social information is expected to be preferred in situations of uncertainty or naivety [8,9,10,46]. This ‘copy when uncertain’ strategy was expected since uncertain environments are a typical feature of bumblebees’ foraging ecology; they gather nectar and pollen from transient, unpredictable floral resources [62,63] that must be rapidly located and exploited. Thus, using social information can provide an effective shortcut, contributing to efficient foraging in bumblebees [37]. Our results also support the idea that in order to produce adaptive benefits, animals should be selective with respect to the individuals from whom they acquire information [8,11,12,14,19,21,64].

### 4.2. Associative Mechanisms Underlying Social Learning

There is no evidence that bumblebees can recognize other individual conspecifics (but see [65]). However, like most animals, they are innately able to recognize their own species from others [25]. Although bumblebees use olfactory and pheromone cues, especially in the darkness of their nest, it is unknown whether bumblebees use olfactory information to identify their own and other species during foraging (but interspecific olfactory eavesdropping in foraging bees is certainly possible; see [66]). The demonstrator bees’ color differences in this study were much greater than many of the subtler visual differences between some bumblebee species. We speculate that our results would be similar if we had used a true heterospecific bumblebee, but if a more dissimilar heterospecific were used, it may have resulted in increased avoidance. It seems plausible that visual information allows for faster information-gathering during foraging situations. Our results suggest bumblebees may be able to visually discriminate between different species of pollinators. Bees in our experiments recognized two types of bees differently paint-marked to simulate heterospecifics while not responding to the discriminating cue when it appeared on non-nectivorous crustaceans (woodlice) or non-animal objects (wooden balls). These results indicate that the color marks of the demonstrators were a feature of specific social cues for the bumblebee observers, rather than merely a condition to elicit an appetitive approach response. Several studies have shown that associative mechanisms can account for seemingly complex behavior in bees and other animals [10,29,46,67]. The selective social information use seen here by bumblebees may also be explained through associative mechanisms. All bumblebees (or any other animal) really have to do is to associate reward with the presence of the compound stimulus of the demonstrator type. However, this association is social by default and sets the stage for flexible and adaptive learning in novel foraging situations.

### 4.3. Suggestions for Future Research

It will be worth exploring, in future experiments, whether bumblebees, and other insects, discriminate between and preferentially utilize some heterospecifics over others in the wild and whether insects can learn more quickly from, or are innately attracted to, ecologically similar species. Further, it would be interesting to know whether solitary insects are able to utilize the reliability of other species as well as social insects, shedding more light on the evolution of these social learning strategies. For the time being, our study provides evidence that an insect has the ability to preferentially utilize differing reward-predictive values of visually discriminable others to guide their subsequent social-information-seeking behavior. Notably, the experimental design here excluded observer bees that appeared to have failed to learn the association in pre-training from subsequent live demonstrator trials in a novel context. In follow-up studies, the experimental design could benefit from exposing these known poor learners to the final live demonstrator phase, or from measuring the success of the pre-training phase only after the live demonstrator test has been completed. Including this measurement in the models as predictor might help to judge the potential biological significance of the results.

## 5. Conclusions

We showed that bumblebees learn to attend to specific types of bees based on their learned value as information providers about reward. Learned information was transferred across foraging contexts, representing a useful pathway for bees to acquire relevant information about unfamiliar floral resources that goes beyond conspecific social information use, extending to heterospecifics.
